# Enhancing corn blight control: synergistic interaction between *Bacillus subtilis* SL-44 and hexaconazole via dual targeting of cell wall and membrane

**DOI:** 10.3389/fmicb.2025.1727044

**Published:** 2026-01-12

**Authors:** Zhongdi Fan, Wenfei Wang, Ji Chen, Haiteng Nie, Wenjie Jia, Jiali Min, Zhansheng Wu, Fei Tian, Xiaojian Chang, Yanhui He

**Affiliations:** 1 Xi’an Key Laboratory of Textile Chemical Engineering Auxiliaries, Engineering Research Center of Biological Resources Development and Pollution Control Universities of Shaanxi Province, Key Laboratory of Textile Dyeing Wastewater Treatment Universities of Shaanxi Province, School of Environmental and Chemical Engineering, Xi’an Polytechnic University, Xi’an, China; 2 Shaanxi Institute of Microbiology, Xi’an, China; 3 Shaanxi Yeenon Selection Co., LTD., Xi'an, China; 4 Xi'an Agricultural Technology Extension Center, Xi'an, China

**Keywords:** *Bacillus* *subtilis*, corn blight, hexaconazole, *Rhizoctonia solani*, synergistic effect

## Abstract

Long-term reliance on chemical fungicides has given rise to issues such as pesticide residue and resistance. Combining fungicides with biological control agents, to reduces the dosage of chemical fungicide has become an important strategy. The synergistic mechanism of *Bacillus subtilis* SL-44 and chemical fungicide hexaconazole in controlling corn blight caused by *Rhizoctonia solani*, were investigated in present study. The results showed that both SL-44 and hexaconazole inhibited *Rhizoctonia solani* growth, and the SL-44 and hexaconazole compound at a 1:9 ratio exhibited significant synergy, with a toxicity ratio of 1.41. Optical and scanning electron microscopy revealed that the combined treatment induced the most severe mycelial damage in *R. solani* compared to individual *Bacillus subtilis* SL-44 or hexaconazole. In addition, the hexaconazole significantly reduced ergosterol content (by 101.63 μg/g), indicating strong inhibition of cell membrane. On the other hand, *B. subtilis* SL-44 caused greater cell wall damage, increasing chitinase and *β*-1,3 glucanase activities by 78.43 U/mL and 0.62 U/mL, respectively. The enhanced efficacy of the combination likely stems from the synergistic effect of these two distinct antimicrobial mechanisms: fungicide action on the cell membrane and biocontrol agent action on the cell wall. Pot experiments confirmed these findings, with the combination achieving an inhibition rate of 72.29%, significantly higher than that of SL-44 (32.58%) or hexaconazole (63.58%) alone. Overall, these results suggest that combining *B. subtilis* SL-44 with hexaconazole is a promising eco-friendly strategy for controlling corn sheath blight, reducing reliance on chemical fungicides while improving disease control efficacy.

## Highlights

- *B. subtilis* and hexaconazole compound at a 1:9 ratio exhibited significant synergy.
- Synergistic inhibitory to *R. solani* was shown by *B. subtilis* and hexaconazole.
- The enhanced efficacy is due to a dual attack on the cell membrane and wall.
- *B. subtilis* and hexaconazole form an eco-friendly approach to control corn blight.

## 1 Introduction

Maize occupies an important position in China’s national economy and agricultural production, serving as a major food source, feed crop, and industrial raw material (Mirsam et al., 2023). In recent years, various diseases have severely damaged maize production areas, resulting in yield losses and quality deterioration (Taheri and Tarighi, 2011). Maize blight, caused by the soil-borne fungus *Rhizoctonia solani* (hereafter abbreviated as *R. solani*), is particularly destructive (Di et al., 2023). It infests the leaf sheaths and stalks near the surface of the maize plant, resulting in cloudy spots. In severe cases, this leads to stalk rot, plant collapse and impeded kernel filling, ultimately causing significant yield losses ranging from 30 to 40% (Song et al., 2011). This disease is widespread in the main maize production areas around the world, and the damage is becoming increasingly serious as maize planting density increases and climate change intensifies (Datta et al., 2022; Dauda et al., 2022).

To date, triazole fungicides are still highly dependent pesticides for the prevention and control of sheath blight in China. As a sterol demethylation inhibitor, hexaconazole exerts bacteriostatic effects by inhibiting the synthesis of ergosterol in the cell membrane of pathogenic fungi (Zhang et al., 2019). It is used to inhibit the growth of fungi on vegetables, fruits and grains, and is widely used as a foliar spray in agriculture (Garcia-Rubio et al., 2021). However, with the growthing attention to the food safety and environmental protection, problems caused by the excessive use of traditional chemical pesticides, such as pesticide residues, resistance generation and ecological imbalance, have received widespread concern (Antypenko et al., 2023). Therefore, the development of low-toxicity, environmentally friendly disease prevention and control strategies has become a current research hotspot (Liu et al., 2018).

In recent years, biocontrol bacteria are regarded as an effective supplement or alternative to chemical control due to their ability to combat pathogenic fungi without developing resistance, their environmental friendliness, and their promotion of ecological health (Li et al., 2013; Shrestha et al., 2016). *Bacillus subtilis* RB4 demonstrated strong antifungal activity, achieving a 60% reduction in the mycelial growth of *F. oxysporum* (Abo-Elyousr et al., 2024). *Bacillus subtilis* SL-44 is a beneficial strain that exhibits significant antagonistic effects against a variety of plant-pathogenic fungi, as determined through preliminary screening (Wang et al., 2024). Previous studies have shown that *Bacillus subtilis* SL-44 has a significant inhibitory effect on *R. solani* through the secretion of enzymes related to disease resistance. Nevertheless, the practical application of biocontrol fungi alone is often limited by inconsistent performance, delayed response, and poor field adaptability, thus failing to achieve satisfactory disease suppression rapidly (Nicolaisen et al., 2018). Consequently, chemical interventions will continue to play a essential role in ensuring the yield of various crops. Abo-Elyousr reported that the combined application of Bion® and *B. subtilis* synergistically enhanced pathogen suppression and diminished disease severity (Abo-Elyousr et al., 2024). it is therefore vital to clarify how to construct synergistic combinations of biocontrol bacteria and chemical fungicides. This research finding will provide guidance for the development of effective resistance management protocols and support the adoption of scientifically sound fungicide practices in agriculture.

However, the success of combination strategies depends on understanding their mutual mechanisms. Although existing studies have delineated the individual mechanisms, such as hexaconazole’s inhibition of CYP51 and its disruptive effects on fungal cell membrane. Yet, how agents like SL-44 and hexaconazole interact synergistically remains unknown. Specifically, the interaction between cell wall disruption and membrane-targeted fungicides to increase pathogen mortality, and whether such interactions can modulate plant defence responses, need to be systematically investigated.

Therefore, this research was designed to decipher the synergistic mechanism between biocontrol agent *Bacillus subtilis* SL-44 and fungicide hexaconazole. We aim to elucidate their combined antagonistic effect against *R. solani*, with the ultimate goals of reducing the hexaconazole dosage, and enhancing overall control efficacy. The synergy was systematically evaluated at an optimal using *in vitro* virulence assays and pot planting test. Our work thus provides a scientific basis for eco-friendly disease management strategies that minimize chemical inputs, effectively bridging fundamental mechanistic research and its practical application in agriculture.

## 2 Materials and methods

### 2.1 Bacterial strains, pathogens, fungicides, and corn varieties

The *Bacillus subtilis* SL-44 was initially screened by the research team from the rhizosphere soil at the early stage. And it has been identified as a biocontrol bacterium with superior spore-producing properties and good environmental adaptability. The bacterium was preserved in the laboratory using glycerol as a protective agent at −80 °C in EP tubes. *R. solani* was kindly provided by the Xue’s Lab from the Shaanxi Institute of Microbiology. The fungicide hexaconazole, which contains 30% active ingredient, was obtained from Jiangsu Sword Agrochemical Co. The corn varieties was Dongdan 6,531 variety, purchased from the Henan Province Dongfeng Seed Industry Co.

### 2.2 Growth of bacteria, fungi and corn

A single SL-44 colony was picked from an LB liquid medium using an inoculation loop and incubated at 28 °C and 180 rpm. Then, 1% of the bacterial solution was aspirated from the LB liquid medium and the sample was continued to be incubated on a shaker until the optical density at 600 nm (OD_600_) reached between 0.8 and 1.0. The sample was then placed in a refrigerator for preservation at −4 °C. The preserved *R. solani* strains were transferred to the centre of a PDA plate and incubated in a dark incubator at 25–28 °C for 5–7 days. Once the new mycelium had expanded to the edge of the colony, the edge of the fungus cake was cut using a sterile perforator (5 mm in diameter), and this was used for subsequent bacterial inhibition experiments and inoculum propagation. Maize was grown using conventional methods: maize seeds were placed in a thermostat at 30 °C with 70% humidity.

### 2.3 Effect of hexaconazole on the growth of *Bacillus subtilis* SL-44 under co-culture conditions

To evaluate the potential toxicity of hexaconazole to *Bacillus subtilis* SL-44, 100 mL of LB liquid medium containing different concentrations of hexaconazole were configured. The concentrations of hexaconazole were set as 0, 0.1, 1, 5 and 10 mg/L. The sterilised medium was inoculated with 1 mL of *Bacillus subtilis* SL-44 fermentation broth. Then they were placed in a shaker at 30 °C and 180 rpm for incubation, and the absorbance was measured at a wavelength of 600 nm at 0, 12, 24, 36, 48 and 72 h to get the growth curve.

### 2.4 Screening of the combination ratio of hexaconazole and SL-44

Following the determination of the IC_50_ of hexaconazole and SL-44 against *R. solani*, concentrations of hexaconazole at IC_50_ = 0.74 mg/L and SL-44 at IC_50_ = 1.38 × 10^8^ CFU/mL were selected for the preparation. According to this concentration, compound agent were prepared with volume ratios of V_(SL-44)_:V_(Hex)_ = 0:10, 1:9, 2:8, 3:7, 4:6, 5:5, 6:4, 7:3, 8:2, 9:1, and 10:0. The synergistic effect was evaluated by calculating the combined virulence based on the inhibition of *R. solani* by the compound agent (Jin et al., 2019). The toxicity ratio (inhibitory ratio) was evaluated using Gu’s method (Gu et al., 2023).


$$\text{Observed effect}\;(\mathrm{Eab})\;(\%)=\frac{\mathrm{Rc}-\mathrm{Rt}}{\mathrm{Rc}}\times 100\%$$

Theoretical effect (Eth) (%)=(Is×Ps + Ih × Ph) × 100%

Inhibitory ratio(IR) $=\frac{\text{Observed effect}\;(\mathrm{Eab})}{\text{Theoretical effect}\;(\mathrm{Eth})\;}$

Rc: Colony radius of the control group. Rt: Colony radius of the treatment group. Is: The actual inhibition rate of SL-44 against *R. solani* at IC_50_. Ps: The proportion of SL-44 in the mixture. Ih: The actual inhibition rate of hexaconazole against *R. solani* at IC_50_. Ph: The proportion of hexaconazole in the mixture.

In this method, an IR < 1 indicates an antagonistic effect, an IR = 1 indicates an additive effect, and an IR > 1 indicates a synergistic effect.

### 2.5 A preliminary investigation of the mechanism of synergistic bacterial inhibition

#### 2.5.1 Optical microscope

A fungal mycelium agar plugs (5 mm in diameter) were aseptically collected from the actively growing margins of fungal colonies and inoculated at the center of PDA plates amended with IC₅₀ of hexaconazole, SL-44, or their combination (V_(SL-44)_:V_(Hex)_ = 1:9). The plates were then incubated at 28 °C until the mycelia had grown to a suitable extent for morphological observation. Representative hyphal segments were then excised from the colony margins (active growth zone) of each treatment group, mounted on microscope slides with sterile water, and gently covered with coverslips to avoid air bubbles. Prepared samples were examined under an optical microscope to document morphological alterations in hyphae (Tian et al., 2024).

#### 2.5.2 Scanning electron microscope (SEM)

The morphology of *R. solani* was observed using a Hitachi Flex SEM1000 scanning electron microscope (SEM). The *R. solani* cultured on a PDA solid medium was punched out using a sterile 5 mm diameter punch and placed in a liquid medium for incubation 72 h. The cultures were then subjected to treatment with the IC_50_ of SL-44, hexaconazole, or the compound mixture exhibiting the highest toxicity ratio for an additional 48 h. The mycelial spheres were then removed in a sterile environment. They were washed 2–3 times with PBS buffer (pH = 7.4), and pelleted by centrifugation. Primary fixation was critical for structural preservation and was carried out using a mixed fixative of 4% paraformaldehyde and 2.5% glutaraldehyde (1:1, v/v). Dehydration was achieved through a sequential ethanol gradient (30, 50, 70, 90, 100%), with 20-min intervals at each concentration, followed by freeze-drying. Before SEM examination, the samples were sputter-coated with a gold layer in a vacuum to ensure optimal conductivity and image contrast. The ultrastructural morphology of *R. solani* from all treatment groups was systematically documented after the 48-h exposure period (Li et al., 2016).

#### 2.5.3 Effect of SL-44 hexaconazole combination on cell wall integrity of *R. solani*

The chitinase activity in *R. solani* cultures was determined according to the method described by Preety et al. (2018). After 48 h of co-cultivation of *R. solani* and *Bacillus subtilis* SL-44, hexaconazole or their combination, 0.5 mL of the supernatant of fermentatiob was collected as the crude enzyme extract and mixed with 0.5 mL of colloidal chitin substrate. The reaction was then incubated in a water bath at 40 °C for 1 h. After incubation, the reaction was terminated by heating in a boiling water bath for 2 min. Subsequently, 1.5 mL of DNS reagent was added, and the solution as heated again in a boiling water bath for 5 min to develop color. After color development, 3 mL of distilled water was added, and the solution was mixed thoughly by shaking. The absorbance of the final solution was measured at 540 nm using a UV spectrophotometer (UC 1600) to get the chitinase activity.

The *β*-1, 3-glucanase activity was determined according to the method described by Zhang et al. (2024). Briefly, 100 μL of a crude enzyme extract from each treatment was mixed with 100 μL of 4 g/L kombucha polysaccharide solution and incubated at 37 °C for 40 min. Subsequently, 1.8 mL of distilled water and 1.5 mL of DNS reagent were added to the mixture. The solution was heated in a boiling water bath for 3 min, then diluted to a final volume of 25 mL with distilled water. The absorbance was measured at 540 nm using a UV spectrophotometer (UC 1600). A standard curve was plotted using glucose treated with the same procedure for quantification.

#### 2.5.4 Effect of SL-44 and hexaconazole combination on membrane permeability of *R. solani*

Ergosterol and malondialdehyde (MDA) contents were measured as indicators of cell membrane integrity and lipid peroxidation extent in *R. solani*, respectively. The MDA content was determined according to a previously described method (Rabab and Reda, 2019). Briefly, mycelial balls from each treatment group were collected and washed three times with PBS buffer (pH 7.4), and homogenized in 5 mL of 10% trichloroacetic acid (TCA). The homogenate was centrifuged at 10,000 rpm and 4 °C for 15 min, and 2 mL of the resulting supernatant was reacted with 2 mL of 0.67% thiobarbituric acid (TBA). The mixture was heated in a boiling water bath for 15 min,. cooled rapidly to room temperature and centrifuged at 5,000 rpm and 4 °C for 10 min. The absorbance of the resulting supernatant was measured at 532 nm, with a blank solution containing 10% TCA and TBA as the reference.

Ergosterol content in the cell membrane of *R. solani* was determined based on Li’s method with minor modifications (Li et al., 2022). Mycelial pellets from different treatments were blotted dry, weighed and subjected to ergosterol extraction by ultrasonication in an organic solvent mixture (V_(methanol)_:V_(chloroform)_ = 2:1). After centrifugation at 8000 rpm (4 C, 10 min), the supernatant was collected and concentrated to near-dryness using a rotary evaporator. The residue was redissolved in 1 mL methanol, filtered through a 0.22 μm organic membrane, and analyzed at 282 nm detection wavelength.

#### 2.5.5 Effect of SL-44 and hexaconazole combination on the damage of *R. solani*

The relative conductivity of the fungal culture was measured to assess cell membrane integrity, following the method of Shen et al. (2021). After 48 h of incubation, the mycelial spheres were collected, rinsed three times with PBS buffer at pH 7.4, and resuspended in fresh PBS, the suspensions were then sonicated at 80 W for 3 min. The relative conductivity of the resulting cell suspension was measured using a conductivity meter.

To evaluate membrane permeability, the release of nucleic acids into the culture medium was determined by measuring absorbance at 260 nm. *R. solani* cultured on a PDA solid medium was punched with a sterile perforator (5 mm diameter) and transferred to a PDA liquid medium for incubation for 3 days. The cultures were then treated with the IC₅₀ concentration of SL-44, hexaconazole, or the most synergistic compound mixture for 48 h. After treatment, 5 mL of the culture solution broth from each group was centrifuged at 4,000 rpm for 5 min, and the absorbance of the supernatant was measured at 260 nm using a spectrophotometer.

#### 2.5.6 Effect of SL-44 and hexaconazole combination on cell membrane leakage of *R. solani*

The extent of cellular leakage in *R. solani* following treatment with SL-44, hexaconazole, and their combination was assessed by measuring the release of soluble proteins and nucleic acids into the extracellular medium.

The soluble protein content was determined using the Coomassie Brilliant Blue staining method. Briefly, 100 μL of the supernatant was mixed with 5 mL of Coomassie Brilliant Blue dye solution and incubated at room temperature for 2–5 min. To prevent potential color fading, the reaction time was strictly controlled to not exceed 10 min. Absorbance was measured at 595 nm using a microplate reader to calculate the soluble protein content (Thermo, Multiskan SkyHigh).

Nucleic acid release was evaluated based on absorbance at 260 nm. Treated mycelium spheres were collected, washed with a PBS buffer (pH 7.4) and resuspended in the same buffer for 6 hours. After centrifugation at 4000 rpm for 10–15 min, 10 μL of the supernatant was diluted with 1 mL of sterile water. Finally, the absorbance of the diluted solution was measured at 260 nm using an enzyme marker to get the Nnucleic acid content (Li et al., 2024).

### 2.6 *In vivo* analysis

The pot experiment was conducted as follows. Fifteen pots were used for maize cultivation, and six seeds were placed in each individual pot. The pot experiment have 4 treatments. CK for control without any additional operations, except for pouring sterile water. Rs for only *R. solani* mycelial suspension treated. Rs + SL-44 for sprayed with *Bacillus subtilis* SL-44 suspension after disease symptoms. Rs + Hex for sprayed with hexaconazole solution at the IC₅₀ concentration. Rs + Mix for the combination of *Bacillus subtilis* Sl-44 and hexaconazole solution at a volum ratio of 1:9. Firstly, all the maize plants except for CK were sprayed with a *R. solani* mycelial suspension advanced when they growths at the three to four leaf stage, causing the corn plants to be infected with the disease. After disease symptoms were established, the plants were treated with various groups. The mycelial suspension was preparated as follows: First, several mycelial plugs were excised from the margin of actively growing fungal colonies using a sterile punch, and then transferred into potato dextrose broth (PDB) for 5 days to facilitate the formation of abundant mycelial pellets. Subsequently, the mycelial pellets were homogenized into fine, uniform fragments using a sterile glass rod under aseptic conditions. Finally, maize leaves were inoculated with the resulting mycelial suspension via sprayer (Kumar et al., 2019). The SL-44 and hexaconazole solution were prepared respectively, by diluting the original solution to the specified IC_50_ concentration calculated based on the inhibitory activity test. Mix for the combination of *Bacillus subtilis* Sl-44 and hexaconazole solutionwere prepared by mixing *Bacillus subtilis* Sl-44 and hexaconazole at a ratio of 1:9 based on the highest toxicity test. Treated applications were repeated every 2 days for a total of three sprays. The experiment included three independent biological replicates, with each treatment consisted of three pots of plants per treatment.

#### 2.6.1 Assessment of plant growth and disease symptoms

The height of each plant was measured from the soil surface to the base of the youngest leaf collar using vernier callipers, with an accuracy of 1 mm. For each treatment, the reported value represents the mean of three independent measurements. To determine biomass, plants were carefully uprooted and rinsed gently with water to remove soil and dust. After surface moisture was blotted dry with absorbent paper, fresh weight was recorded. Samples were then oven-dried at 90 °C to constant weight to obtain dry weight. Disease severity was quantified by measuring the lesion area on maize leaves using Image-Pro Plus software (Araújo et al., 2022). The calculated lesion area was used to evaluate the disease status across treatment groups.

#### 2.6.2 Determination of maize antioxidant enzyme activities

A total of 0.2 g of fresh plant leaf tissue was homogenized in a mortar with 2 mL of phosphate buffer until fully disrupted. The homogenate was centrifuged (4 °C, 10,000 rpm for 20 min), and the supernatant was collected as the crude enzyme extract for subsequent assays.

Plant superoxide dismutase (SOD) enzyme activity was determined by the nitrogen blue tetrazolium (NBT) method. The reaction mixture contained 1 mL of phosphate buffer solution (pH 7.8), 0.1 mL of 75 μM NBT, 0.1 mL of 10 mM sodium lactate, 0.1 mL of 10 mM nitroacetic acid, and 0.1 mL of the enzyme extract. After incubation at 37 °C for 20 min, the reaction was terminated, and absorbance was measured at 580 nm.

Peroxidase (POD) activity was assessed by monitoring the oxidation of guaiacol at 470 nm. The reaction system consisted of 1 mL of phosphate buffer (pH 6.0), 0.1 mL of 20 mM guaiacol, 0.1 mL of 30 mM H₂O₂, and 0.1 mL of enzyme extract. The reaction proceeded at 25 °C for 5 min before absorbance was recorded.

Catalase (CAT) activity was measured based on the decomposition rate of H₂O₂, indicated by the decrease in absorbance at 240 nm. The reaction mixture included 1 mL of phosphate buffer (pH 7.0), 0.1 mL of 50 mM H₂O₂, and 0.1 mL of enzyme extract. The change in absorbance was determined after 1 min of reaction at 25 °C (Padró et al., 2021).

### 2.7 Statistical analysis

The combined effect of SL-44 and hexaconazole was evaluated by comparing the observed control efficacy with the expected efficacy calculated using the Colby method (Peng et al., 2017). A positive difference indicates a synergistic interaction, whereas a negative or zero value denotes an antagonistic or additive effect, respectively. The magnitude of this value reflects the strength of the interaction.

All data are presented as the mean ± standard error (SE) of three independent replicates. Statistical analyses were conducted using SPSS 25.0 (SPSS Inc., Chicago, IL, USA). Significant differences among treatment groups were determined by one-way analysis of variance (ANOVA) followed by Duncan’s multiple range test, with a significance level of *p* < 0.05.

## 3 Results

### 3.1 Synergistic effect and compatibility of *Bacillus subtilis* SL-44 with hexaconazole on *R. solani*

**Figure 1:**
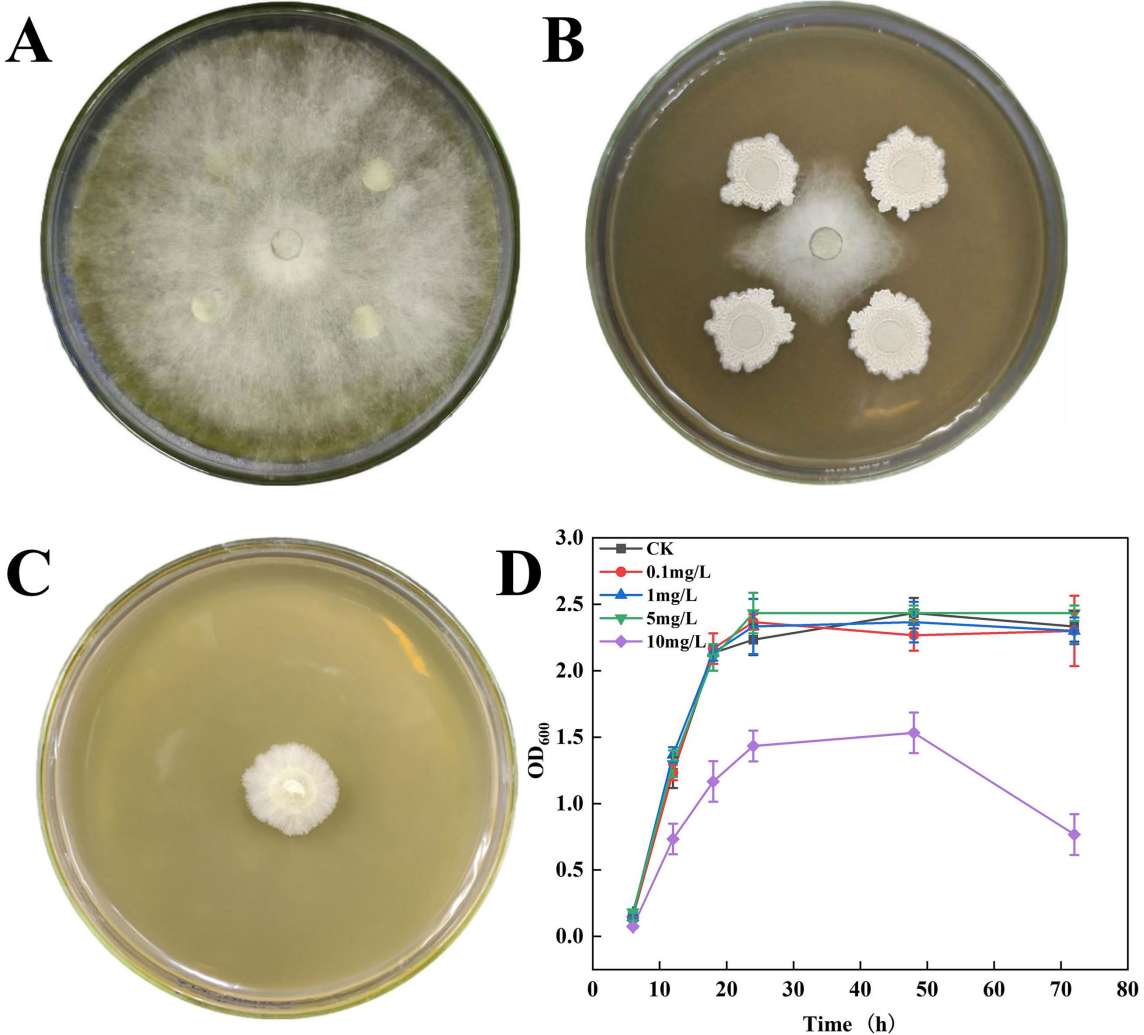
Antifungal activity of SL-44 and hexaconazole against *R. solani*. **(A)** Blank control group. **(B)** *R. solani* inhibited by SL-44. **(C)** *R. solani i*nhibited by hexaconazole. **(D)** Growth curve of SL-44 under different hexaconazole concentrations.

The inhibitory effects of SL-44 and hexaconazole on *R. solani* were investigated individually (Figure 1). After 5 days of incubation, the blank control group showed normal fungal growth (Figure 1A), in contrast to the significant inhibition observed in the presence of SL-44 (Figure 1B) or hexaconazole (Figure 1C). Compatibility assays further revealed that SL-44 tolerated low concentrations of hexaconazole (Figure 1D). Specifically, no adverse effect on SL-44 growth was detected at hexaconazole concentrations up to 5 mg/L, whereas marked inhibition became evident at 10 mg/L.

### 3.2 IC₅₀ of SL-44 and hexaconazole against *R. solani*

**Figure 2:**
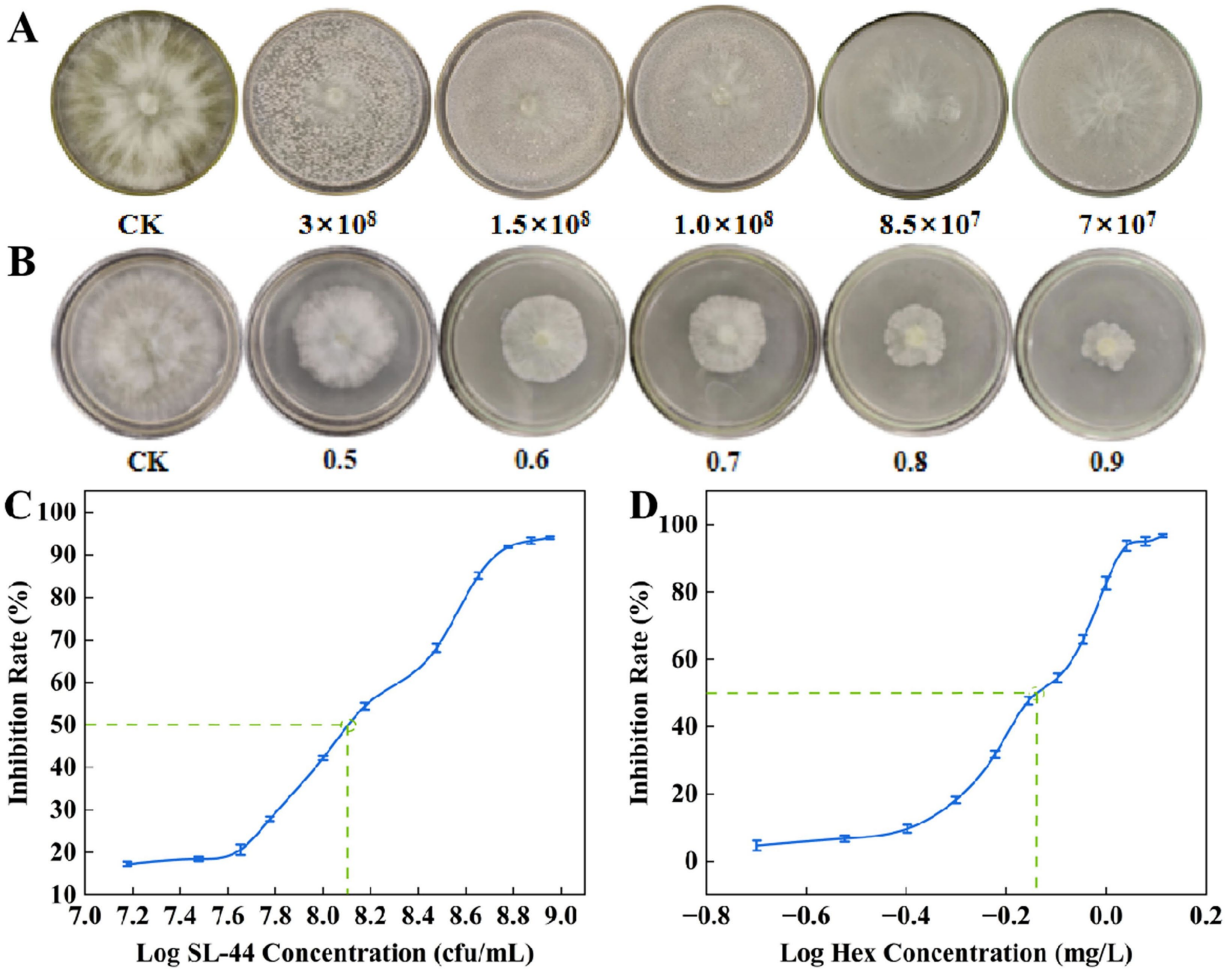
Inhibitory activity and virulence regression of SL-44 and hexaconazole against *R. solani*. Inhibition activity of *R. solani* by **(A)** SL-44 and **(B)** hexaconazole (Hex) at different concentrations. Virulence regression curves of **(C)** SL-44 and **(D)** hexaconazole (Hex) calculated based on the inhibition activity.

To compare the individual inhibitory effects of SL-44 and hexaconazole on *R. solani*, their half-maximal inhibitory concentration (IC_50_) was determined. As shown in Figures 2A,B, SL-44 (7 × 10^7^ cfu/mL) and hexaconazole (0.5 mg/L) achieved inhibition rates of 28.05 and 18.18%, respectively, at low concentrations. The inhibition increased with concentration, reaching 67.07% for SL-44 at 3 × 10⁸ cfu/mL and 65.91% for hexaconazole at 0.9 mg/L. Based on the regression equations of inhibition rate against log concentration, the IC₅₀ values were calculated to be 1.38 × 10⁸ cfu/mL for SL-44 (y = 55.163x – 399.1) (Figure 2C) and 0.74 mg/L for hexaconazole (y = 186.22x + 74.205) (Figure 2D).

### 3.3 Synergistic antagonistic activity of SL-44 and hexaconazole against *R. solani*

The antagonistic activity of SL-44 and hexaconazole combinations against *R. solani* is summarized in Table 1. A synergistic effect (toxicity ratio > 1) was observed across most mixing ratios. The highest toxicity ratio (1.41) was achieved at a V_(SL-44)_:V_(Hex)_ ratio of 1:9, indicating the strongest synergy. As the proportion of hexaconazole decreased, the toxicity ratio gradually declined. The synergistic effect was weakest at a ratio of 9:1, with a toxicity ratio of 0.95, indicating antagonism. Consequently, the 1:9 ratio was selected for subsequent metabolomics analysis.

**Table 1:** Toxicity ratio of combinations against *R. solani* under different concentration ratios of SL-44 and hexaconazole.

V_(SL-44)_:V_(Hex)_	Colony radius (cm)	Observed effect (Eab) (%)	Theoretical effect (Eth) (%)	Inhibitory ratio (IR)
CK	7.0	0	0	0
1:9	2.2 ± 0.05d	69.04 ± 0.82a	48.86	1.41 ± 0.01a
2:8	2.6 ± 0.26 cd	62.86 ± 3.78ab	49.14	1.28 ± 0.07ab
3:7	2.8 ± 0.30bc	60.00 ± 4.29bc	49.43	1.21 ± 0.08bc
4:6	2.9 ± 0.21bc	59.05 ± 2.98bc	49.71	1.19 ± 0.05bc
5:5	3.0 ± 0.26bc	57.14 ± 3.78bc	50.00	1.14 ± 0.07bcd
6:4	3.2 ± 0.26abc	54.28 ± 3.78bcd	50.28	1.08 ± 0.07cde
7:3	3.2 ± 0.40abc	54.29 ± 5.72bcd	50.57	1.07 ± 0.11cde
8:2	3.4 ± 0.50ab	51.43 ± 7.14 cd	50.85	1.01 ± 0.14de
9:1	3.6 ± 0.36a	48.57 ± 5.15d	51.14	0.95 ± 0.10e

Data are presented as mean ± standard error (SE; *n* ≥ 3). Different lowercase letters above bars indicate statistically significant differences among treatments according to one-way ANOVA (*p* < 0.05).

### 3.4 Effect of combined treatment on *R. solani* mycelium

**Figure 3:**
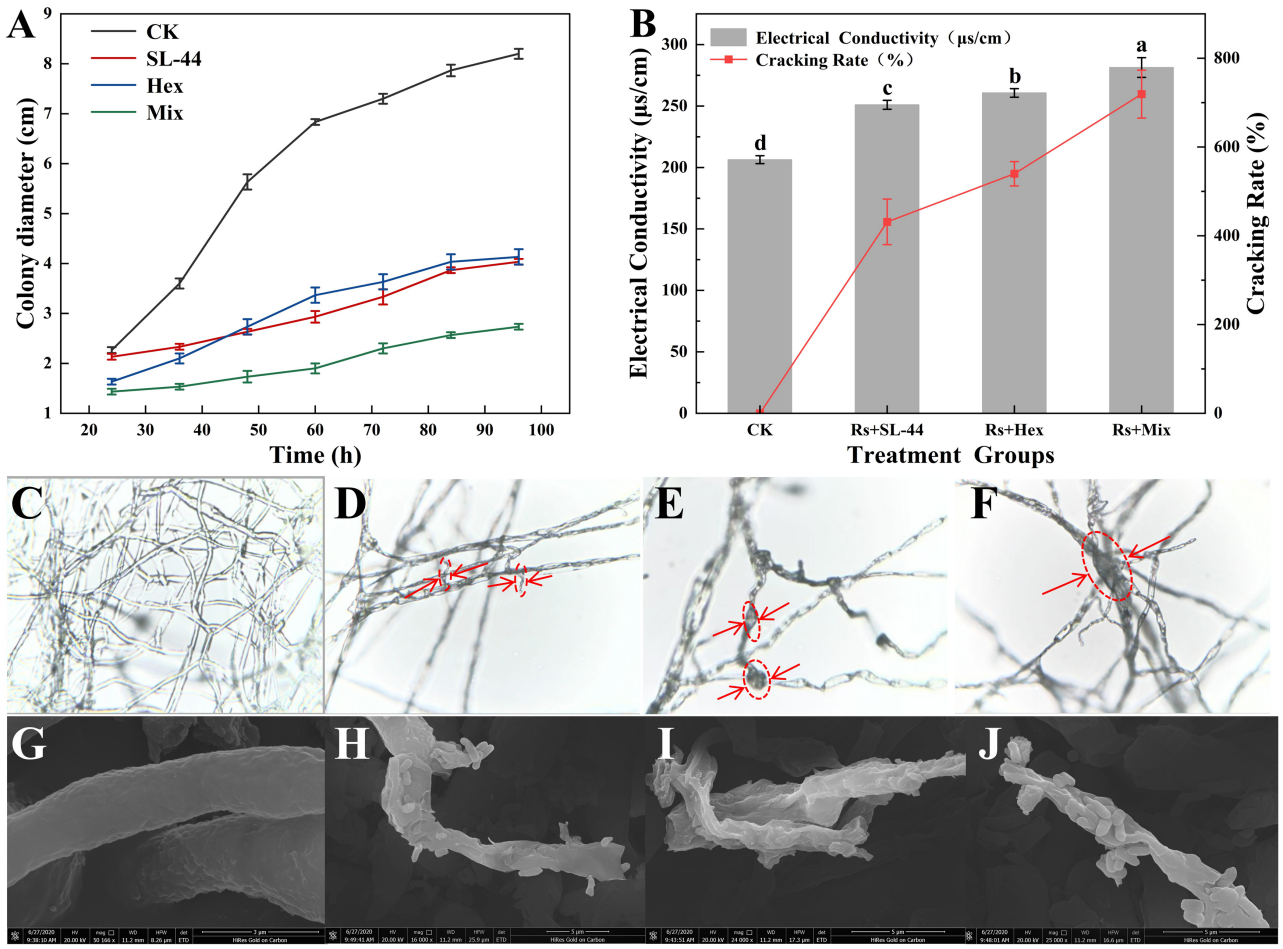
The inhibitory activity of SL-44, hexaconazole (Hex), and their mixture (Mix) against *R. solani*. **(A)** Mycelial colony diameter growth kinetics under different treatment. **(B)** Cell membrane integrity index. **(C–F)** Optical micrographs of hyphae after treatment: **(C)** for CK (untreated control), **(D)** for SL-44 treated, **(E)** for Hex treated, and **(F)** for Mix treated. **(G–J)** Corresponding SEM images of hyphal ultrastructure for CK, SL-44, Hex, and Mix treated.

The effects of SL-44, hexaconazole, and their combination on the mycelial growth of *R. solani* were systematically evaluated (Figure 3A). Measurements of hyphal radius showed that during the early stage (24–44 h), the hexaconazole treated group exhibited maintained consistently slower growth than the SL-44 treated group. By 44 h, both groups has reached a similar hyphal radius of 2.5 cm. However, from 44 to 96 h, the SL-44 treatment group remained consistently slower growth compared to hexaconazole treatment group. In contrast, the combined treatment group (Mix) resulted the smallest mycelial radius throughout the entire incubation period.

These results suggest that hexaconazole serves as the primary inhibitory agent in the early stages, likely by inhibiting ergosterol biosynthesis and disrupting the integrity of the fungal cell membrane. As the culture progressed, the inhibitory role of SL-44 became more pronounced, possibly through competitive exclusion and the production of antimicrobial metabolites, gradually becoming the dominant factor in suppressing mycelial growth.

Morphological observations under optical and scanning electron microscopy further revealed structural alterations in the hyphae of *R. solani* across treatments. Untreated hyphae exhibited healthy growth with uniform branching and smooth, continuous connections between branches and main hyphae (Figures 3C,G). In contrast, treatment with SL-44 resulted in surface damage, depressions, and irregular morphological changes, leading to thinner, sparser hyphae and reduced branching (Figures 3D,H). Hexaconazole treatment induced the formation of “vacuole-like structures” on the hyphal surface (Figures 3E,I). The combined treatment (Mix) caused the most severe damage, including widespread hyphal thinning, lysis, and structural collapse (Figures 3F,J). This consistent with the growth inhibition results above.

### 3.5 Effect of combination on cell wall damage of *R. solani*

**Figure 4:**
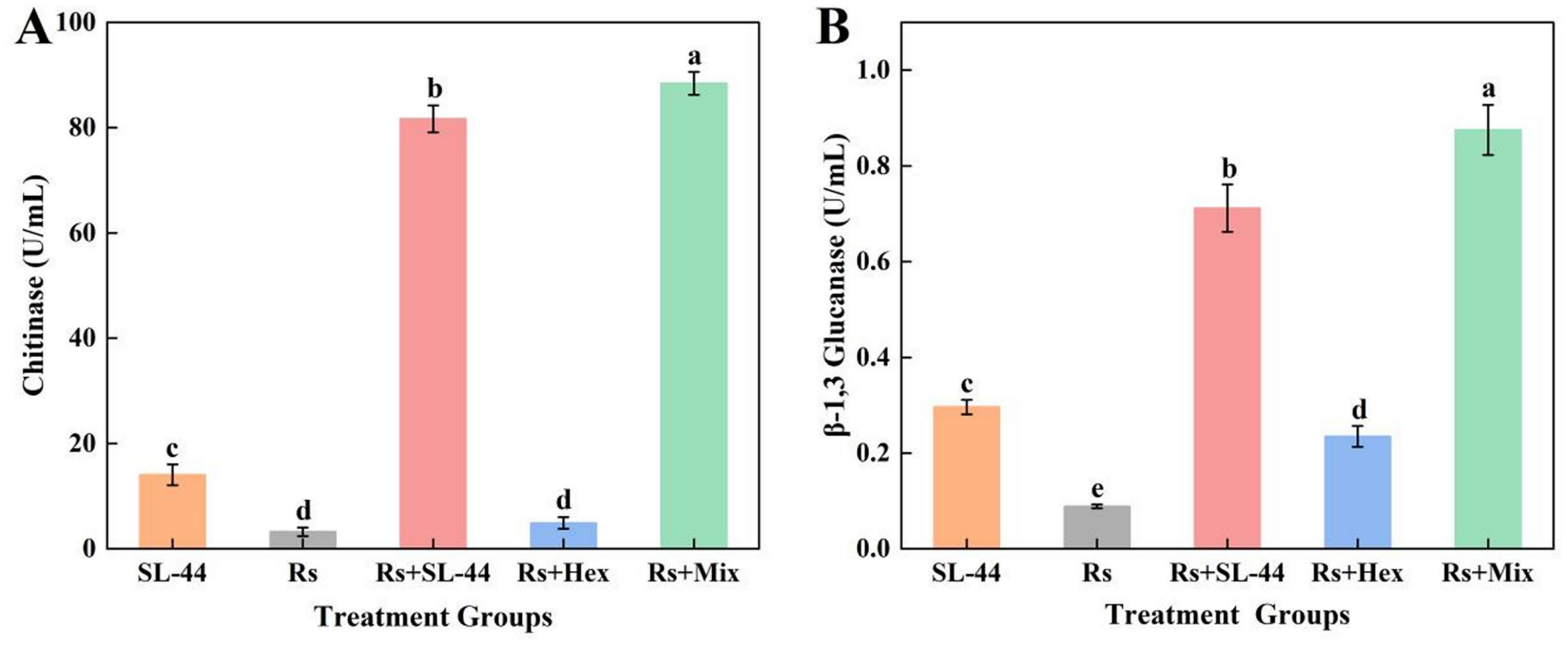
Effect of different treatments on *R. solani* cell wall-degrading enzyme activities. **(A)** Chitinase activity. **(B)** *β*-1,3-glucanase activity.

Chitinase and *β*-1,3-glucanase are two key cell wall degrading enzymes involved in plant defense and biological processes. They specifically target chitin and β-1,3-glucan, the major structural polysaccharides of the fungal cell wall, disrupting their integrity and leading to growth inhibition or direct mycelial lysis. To elucidate the antifungal mechanism of the biocontrol bacteria SL-44 and its synergistic interaction mechanism with hexaconazole, the activities of chitinase and β-1,3-glucanase in *R. solani* were measured after 48 h of exposure to SL-44, hexaconazole and their combination. The results indicated that SL-44 induced significantly greater cell wall damage in *R. solani* than hexaconazole, suggesting that its primary mode of action involves the degradation of the fungal cell wall. The combined treatment further enhanced this damaging effect, resulting in the most severe cell wall disruption among all groups. Specifically, the chitinase and β-1,3-glucanase avtivities increased by 8.27% (Figure 4A) and 23.07% (Figure 4B), respectively, compared to SL-44 alone. It is plausible that the initial cell wall degradation by SL-44 facilitated the permeation of hexaconazole into the fungal cell, thereby intensifying the overall damage to the cell wall and strengthening the combined inhibitory effect on *R. solani*.

### 3.6 Effect of combination on cell membrane damage of *R. solani*

**Figure 5:**
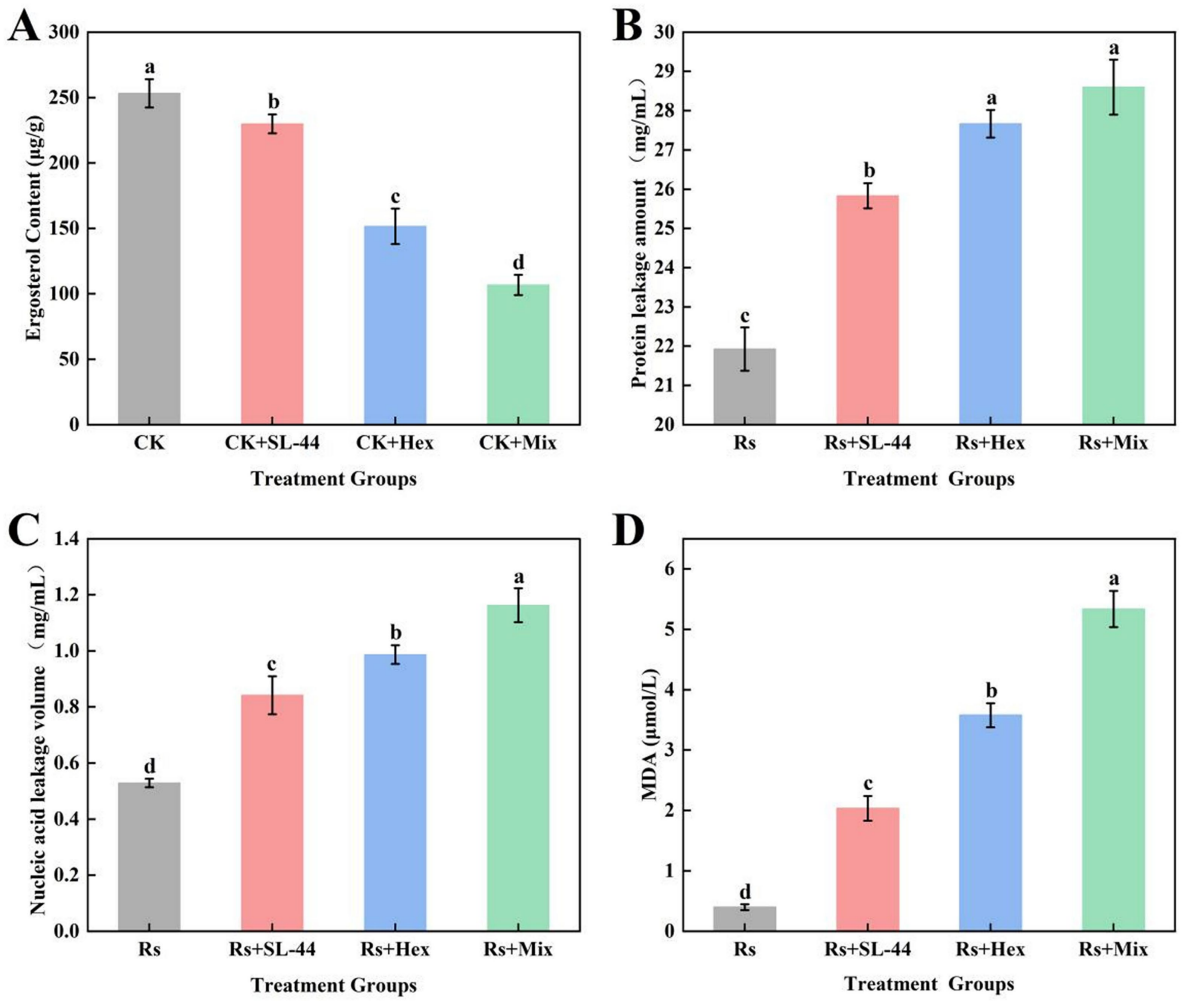
Assessment indicators changes of cell membrane damage of *R. solani* caused by SL-44, hexaconazole (Hex), and their mixtures (Mix): **(A)** Ergosterol content; **(B)** Soluble protein released amount; **(C)** Nucleic acid leakage amount; **(D)** MDA content.

Ergosterol is a key component of the fungal cell membrane, and its content can serve as an indicator of the extent of membrane damage caused by hexaconazole in *R. solani*. Additionally, soluble proteins, nucleic acids, and malondialdehyde (MDA) also serve as important indicators for reflecting membrane integrity and oxidative stress status. Therefore, to systematically evaluate membrane integrity and oxidative stress, the ergosterol content (Figure 5A), extracellular soluble protein release (Figure 5B), nucleic acid leakage (Figure 5C), and MDA content were systematically quantified (Figure 5D) after 48 h of treatment with SL-44, hexaconazole, or their combination. Compared to the SL-44 alone group, the hexaconazole-treated group showed a significant reduction in ergosterol content by 34.05%, indicating strong inhibition of its biosynthesis. Concurrently, soluble protein release, nucleic acid leakage and MDA content significantly increased by 7.10, 17.86 and 75.77%, respectively. These results demonstrate that hexaconazole induces severe lipid peroxidation, disrupts membrane structure, enhences permeability, and promotes the leakage of intracellular components, thereby effectively suppressing *R. solani* growth.

Notably, the combined treatment caused more pronounced membrane damage effects on *R. solani* than hexaconazole alone. The protein leakage further increased by 3.36%, nucleic acid leakage by 17.83%, and MDA content significantly increased by 49.15%. This synergistic effect led to a pronounced decline in cell viability, with the combined treatment exhibiting a relative conductivity 1.39 times higher and a cell lysis rate 1.97 times higher than those in the hexaconazole only treatment (Figure 3B). These results indicate that hexaconazole increases membrane permeability by inhibiting ergosterol synthesis, thereby facilitating the penetration of antimicrobial metabolites derived from SL-44. This process accelerates the degradation and leakage of intracellular contents, ultimately intensifying the hyphal lysis to a significant extent.

### 3.7 Effect of combination on the control of corn blight

**Figure 6:**
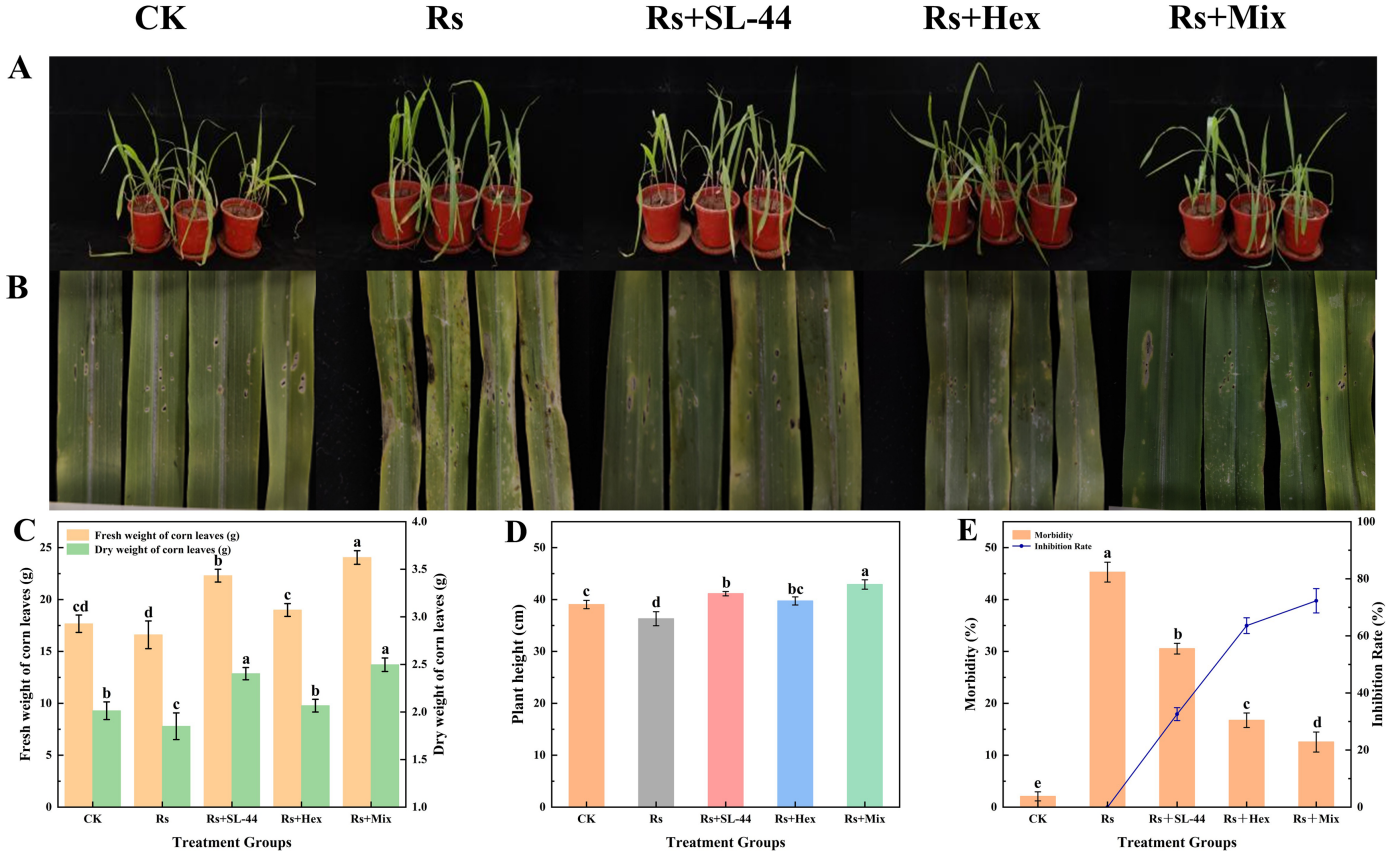
Effects of mixtures (Mix) of SL-44 and hexaconazole (Hex) on the severity of maize sheath blight and plant growth. **(A)** The overall condition of corn plants. **(B)** The leaf disease symptoms. **(C)** Fresh and dry weight of corn leaves for various treatments. **(D)** Plant height of corn plant. **(E)** Infection status.

In pot experiments, maize plants infected with sheath blight were treated with SL-44, hexaconazole, and their combination. To evaluate the efficacy of individual and combined applications in controlling the disease (Figure 6). Observation of the leaf infection status revealed that, compared with the control group (CK), the incidence rate of corn leaves increased from 2.07 to 45.28% after the application of *R. solani*. Application of SL-44, hexaconazole, and their combination reduced the disease incidence from 45.28 to 30.53%, 16.74, and 12.54%, respectively. The control efficacy of SL-44 alone was 32.58%, while hexaconazole alone achieved 63.58%. The combined treatment showed a significantly higher efficacy of 72.29% (Figure 6E). Additionally, compared to the control (CK), the combined treatment resulted in notable increases in leaf dry weight, fresh weight, and plant height by 23.84, 34.11, 9.91%, respectively, outperforming all individual treatments (Figures 6C,D). In conclusion, the combined treatment not only displayed remarkable inhibitory effects against maize sheath blight but also promoted crop growth.

### 3.8 Effect of SL-44 and hexaconazole on defense enzymes activities in maize leaves

**Figure 7:**
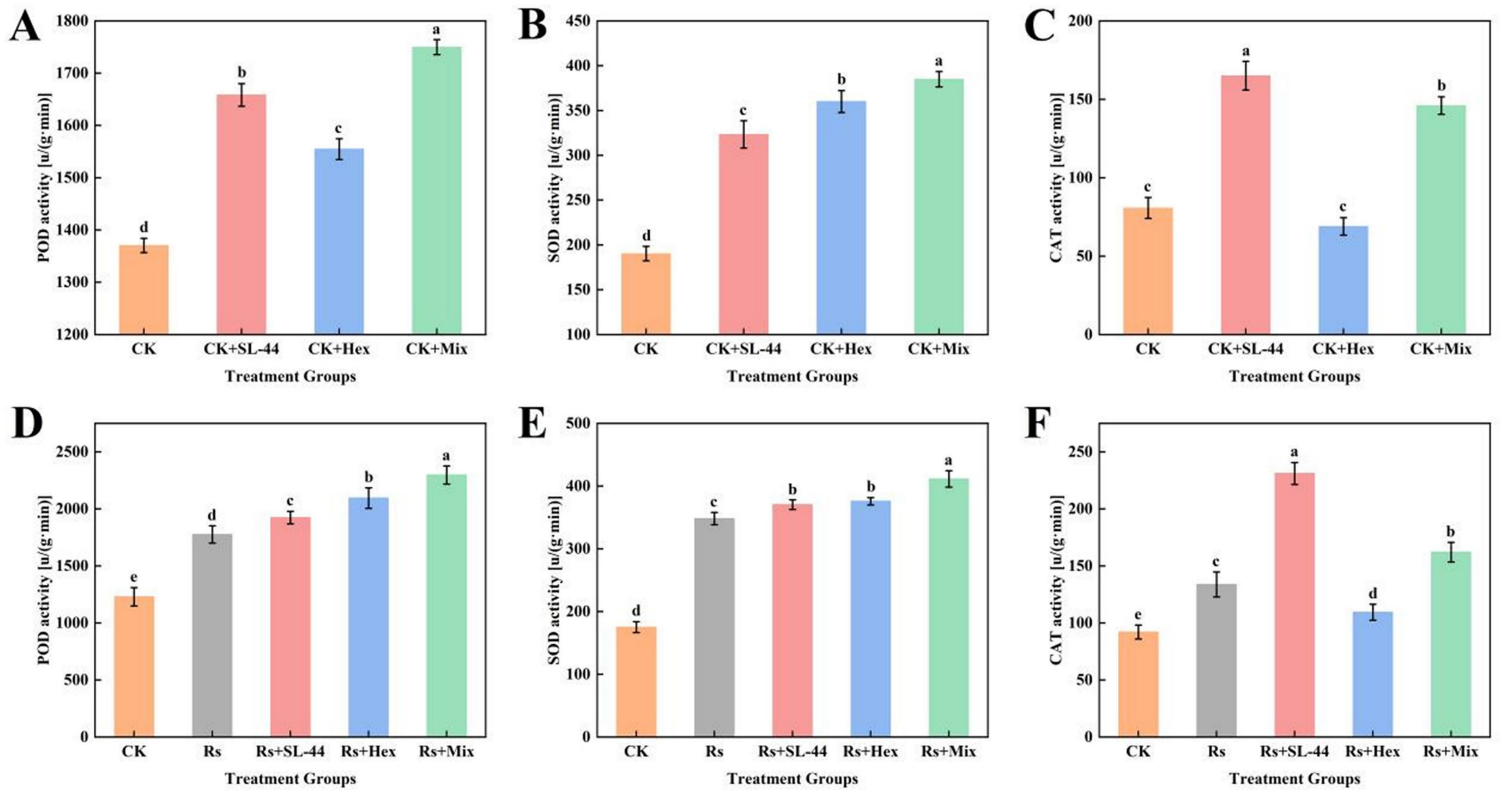
Effects of mixtures (Mix) of SL-44 and hexaconazole (Hex) on the metabolism of reactive oxygen in maize leaves. **(A–C)** POD, SOD, and CAT enzyme activity before application of *R. solani*. **(D–F)** POD, SOD, and CAT enzyme activity after application of *R. solani*.

The activities of antioxidant enzymes, including SOD, POD, and CAT, are key indicators of plant defense responses under biotic stress. To evaluate the effect of the treatments on plant defense capacity, the activity of these three defense-related enzymes (POD, SOD and CAT) were analysed before and after the application of *R. solani.* Before *R. solani* infection, the combined treatment with SL-44 and hexaconazole significantly enhanced the activities of POD and SOD compared to the control (Figures 7A,B). CAT activity in the co-treatment group, although lower than that in the SL-44 alone group, remained higher than that in the hexaconazole-only treatment (Figure 7C). After *R. solani* infection, all treatment groups showed a significant increase in POD, SOD, and CAT activities relative to pre-infection levels. The combined treatment resulted in higher POD and SOD activities than either individual treatment (Figures 7D,E), while CAT activity was lower than in the SL-44 alone group but higher than in the hexaconazole-only group (Figure 7F). Overall, the combined application of SL-44 and hexaconazole enhanced the activities of POD, SOD, and CAT more effectively than hexaconazole applied alone.

## 4 Discussion

The extensive use of fungicides has led to their accumulation in crops and soils, with high concentrations of triazole fungicides adversely affecting microbial communities. Integrating chemical fungicides with biocontrol agents has emerged as a widely accepted strategy to broaden the antimicrobial spectrum, enhance disease control efficacy, and reduce resistance risks (You et al., 2016), as evidenced in crops like tomato (Huang et al., 2025) and coriander (Abdelrhim et al., 2023). In this study, we demonstrated that the combined application of *Bacillus subtilis* SL-44 and hexaconazole effectively controlled maize sheath blight caused by *R. solani*. We hypothesize that this enhanced efficacy stems from a synergistic interaction between their distinct antifungal mechanisms.

As a prerequisite for combined application, the compatibility between *Bacillus subtilis* SL-44 and hexaconazole was first evaluated. Consistent with previous reports on the concentration-dependent effects of fungicides on biocontrol bacteria (Xu et al., 2022), SL-44 exhibited robust growth even at relatively high hexaconazole concentrations (Figure 1D), indicating good biocompatibility. Subsequently, we investigated the optimal combination ratio and found that most co-treatment groups outperformed individual treatments. In particular, the combination with a volume ratio of V_(SL-44)_:V_(Hex)_ = 1:9 showed the highest toxicity ratio (1.41). However, the inhibitory effect gradually decreased as hexaconazole concentration decreased (Table 1), a trend consistent with earlier findings of Gu et al. (2023), who reported the strongest suppression of pathogen Pn006 at V_(Czk1)_:V_(Genkang)_ = 2:8, with efficacy declining as Genkang concentration decreased. This phenomenon can be attributed to the fact that at the 1:9 ratio, hexaconazole—which directly disrupts fungal membrane integrity—acts as the primary inhibitory agent, while SL-44 enhances its efficacy through auxiliary mechanisms such as inducing the synthesis of chitinase and *β*-1,3-glucanase (Kumari et al., 2024). As the hexaconazole concentration decreases, its targeted inhibition weakens, and the biocontrol efficacy of SL-44 (e.g., antimicrobial metabolite production) may be insufficient to fully compensate, leading to reduced synergy. Therefore, the V_(SL-44)_:V_(Hex)_ = 1:9 combination was selected for subsequent mechanistic studies.

To evaluate the morphological effects of different treatments on *R. solani*, we observed hyphal structures using both optical and scanning electron microscopy (SEM). Similar to the SEM observations of *Botryosphaeria dothidea* mycelia treated with the *Bacillus velezensis* LMY3-5 (Ren et al., 2025), our study also found that SL-44 induced obvious morphological damage to *R. solani* hyphae, including hyphal curling, fragmentation (Figure 3D), and surface damage with depressions (Figure 3H). In contrast, hexaconazole treatment resulted in vacuole-like structures, suggesting cell membrane disruption (Figures 3E,I). Kang et al. reported that metconazole induced *F. culmorum* hyphal ultrastructural abnormalities under SEM, including irregular hyphal swelling and cytoplasmic inclusion formation (Kang et al., 2001). Our SEM data align with this core observation: hexaconazole treatment also caused morphological deformities in *R. solani* mycelia (e.g.vacuole-like structures), confirming that triazole fungicides universally disrupt fungal cell membrane integrity via ergosterol biosynthesis inhibition. The combined treatment caused more extensive damage, with pronounced longitudinal wrinkling and depression, affecting nearly all hyphal segments affected (Figures 3F,J). Similar to the morphological damage reported in *Aspergillus fumigatus* under combined stress (Meng et al., 2023), the *Staphylospora grisea* mycelium treated with the combined strategy showed extensive depressions covering nearly the entire hyphal segment and was characterized by dense surface folds. In some areas, mycelium breakage occurred, and the prevalence and severity of the damage were significantly enhanced. This pronounced deterioration reflects the synergistic action of the biocontrol agent and the fungicide.

Similarly, damage to the pathogen cell wall was reflected in the activities of cell wall-degrading enzymes. SL-44 treatment significantly increased the activities of chitinase and *β*-1,3-glucanase (Figures 4A,B), which degrade key structural components of the fungal cell wall (Hong et al., 2017), consistent with previous studies (Yin et al., 2023). The combined treatment further enhanced these levels of the detected indicators in *R. solani*, likely due to complementary actions: SL-44-mediated cell wall disruption may facilitate hexaconazole penetration, and amplifying its membrane-damaging effects. This “dual attack” strategy was further supported by the higher relative electrical conductivity and hyphal lysis rate in the co-treatment group (Figure 3B). A similar synergistic mechanism has been reported in medical antifungal therapy, where the combination of echinocandins and azoles casesed severe mycelium damage, as evidenced by electron microscopy image (Tashiro et al., 2020). Our study extends this concept to agricultural fungal pathogens, offering a strategy for developing highly effective and low-toxicity composite fungicides. Damage to the pathogen cell membrane was further supported by physiological and biochemical assays. Ergosterol, an essential component of the fungal cell membrane, plays an indispensable role in maintaining membrane structural integrity and functional stability (Cao et al., 2024). Hexaconazole significantly reduced ergosterol content in *R. solani* (Figure 5A). Concurrently, leakage of intracellular components, including soluble protein (Figure 5B), nucleic acids (Figure 5C), and malondialdehyde (MDA) (Figure 5D), increased markedly. These findings align with studies demonstrating that triazole fungicides primarily impair fungi by targeting and disrupting the cell membrane (Draskau et al., 2019). The combined treatment resulted in the lowest ergosterol content and the highest level of cellular leakage, indicating strong synergy and accelerated hyphal death.

Critically, these *in vitro* findings were validated in a pot experiment. The combined application significantly reduced the disease severity with the following gradient: Rs > CK > SL-44 > hexaconazole > combined treatment (Figure 6E), clearly demonstrating the superiority of the integrated control. The moderate efficacy of SL-44 alone may be attributed to its reliance on population expansion and gradual accumulation of antimicrobial compounds, coupled with limited colonization efficiency on leaves—a pattern consistent with previous studies (Ramzan et al., 2016),. In contrast, hexaconazole provides rapid cell membrane disruption and short-term suppression but weaker long-term control, as also reported in studies of hexaconazole against *Botrytis cinerea* (Liu et al., 2024). Furthermore, the combined treatment significantly enhanced the activities of POD and SOD in maize plants compared to individual treatments, indicating a more effective activation of plant systemic defenses. This group also exhibited the lowest disease incidence, along with significantly increased plant height, fresh weight, and dry weight, demonstrating a virtuous cycle of “enhanced growth–elevated resistance–reduced disease” and achieving synergistic effects in both disease control and growth promotion. These results not only confirm the in vitro observations but also underscore the practical effectiveness of this integrated strategy in more complex and biologically relevant systems. The enhanced disease suppression achieved with the combined treatment suggests that the chemical fungicides dosage can be reduced without compromising efficacy, thereby mitigating environmental and health risks.

## 5 Conclusion

In this study, the antibacterial activity and mechanism of action of *Bacillus subtilis* SL-44 and hexaconazole against *R. solani* were systematically investigated through in vitro and *in vivo* experiments. The *Bacillus subtilis* SL-44 primarily targets the cell wall of *R. solani*, which can induce structural abnormalities and increasing cell wall permeability. This is evidenced by elevated activities of chitinase and *β*-1,3 glucanase, destruction of cell wall structure, and enhanced leakage of intracellular compound such as soluble proteins and nucleic acid. In contrast, hexaconazole causes damage to the cell membrane by inhibiting the biosynthesis of ergosterol in *R. solani*. This disruption directly results in the leakage of osmotic substances such as malondialdehyde (MDA), and ultimately results in the loss of membrane function. Experiments have confirmed that the combination of SL-44 and hexaconazole exhibits significantly superior efficacy in eradicating *R. solani* than the individual treatments, demonstrating a substantial synergistic effect. This synergistic effect stem from complementarity targeting of pathogen’s cellular structures by the two agents: the cell wall damage caused by SL-44 provides a convenient channel for hexaconazole to enter the bacterial cells, while the cell membrane induced by hexaconazole exacerbates the leakage of intracellular substances triggered by SL-44, forming a “dual strike” effect. Furthermore, pot experiments confirmed that the combination treatment effectively reduced disease severity in maize caused by *R. solani* infection. This also outperformed individual treatments in promoting plant growth—as reflected in fresh weight, dry weight, and plant height—supporting the practical value of this synergistic strategy under realistic agricultural conditions. In conclusion, by simultaneously targeting different cellular structures of the pathogen, this combination approach of SL-44 and hexaconazole not only enhances the control efficacy, but also reduce the risk of the pathogen developing drug resistance, providing a sustainable alternative to sole reliance on chemical fungicides.

## Data Availability

The raw data supporting the conclusions of this article will be made available by the authors, without undue reservation.
